# 环状RNA分子circ_0007766通过上调细胞周期相关蛋白Cyclin D1/CyclinE1/CDK4的表达促进肺腺癌细胞增殖

**DOI:** 10.3779/j.issn.1009-3419.2019.05.03

**Published:** 2019-05-20

**Authors:** 帅 张, 文佳 夏, 高超 董, 维章 徐, 明 李, 林 许

**Affiliations:** 210009 南京，江苏省肿瘤医院 & 江苏省肿瘤防治研究所 & 南京医科大学附属肿瘤医院，胸外科；江苏省恶性肿瘤分子生物学及转化医学重点实验室 Department of Thoracic Surgery, Jiangsu Cancer Hospital & Jiangsu Institute of Cancer Research & The Affiliated Cancer Hospital of Nanjing Medical University, Jiangsu Key Laboratory of Molecular and Translational Cancer Research, Nanjing 210009, China

**Keywords:** 肺腺癌, 环状RNA/circRNA, circ_0007766, Lung adenocarcinoma, circRNA, circ_0007766

## Abstract

**背景与目的:**

环状RNA（circular RNA, circRNA）是有别于传统线性RNA的一类新型非编码RNA（non-coding RNA, ncRNA），越来越多的研究提示circRNA可作为诸多恶性肿瘤的生物学标志物，并成为治疗的潜在靶标。因此从circRNA角度挖掘肺腺癌新型分子靶标，将有助于揭示肺腺癌发生发展新机制，并为临床诊疗提供新思路。本研究针对前期筛选出的一个肺腺癌组织高表达circRNA即circ_0007766进行体外生物学功能验证和分析，从而对circ_0007766促进肺腺癌增殖的相关机制进行初步探索。

**方法:**

首先通过qPCR检测考察肺腺癌细胞中circ_0007766的表达水平，然后采用siRNA干扰circ_0007766的表达，通过CCK8法、划痕修复、PI单染和AnnexinV/PI双染方法检测circ_0007766沉默表达对肺腺癌细胞的增殖、周期和凋亡作用的影响。此外，我们还采用qPCR和western blots方法初步研究了circ_0007766在肺腺癌细胞中的生物学作用机制。

**结果:**

qPCR检测表明肺腺癌细胞株中表达有circ_0007766，在SPCA-1细胞中干扰circ_0007766表达，发现细胞增殖和迁移能力受到了抑制，细胞周期发生了G_0_/G_1_期阻滞，而细胞凋亡则不受影响。circ_0007766的缺失并不影响其亲本基因ERBB2的mRNA表达，而对eIF4A3调控的细胞周期相关基因Cyclin D1/Cyclin E1/CDK4的mRNA和蛋白水平均有影响。

**结论:**

本研究通过体外功能学研究，发现circ_0007766可能对肺腺癌细胞增殖、迁移具有促进的作用，进一步的分子机制研究发现circ_0007766可上调细胞周期关键蛋白Cyclin D1/Cyclin E1/CDK4的表达，进而促进肺腺癌恶性增殖。本研究以circRNA为视角，将对肺腺癌发生发展机理及预后判断提供新线索，为临床治疗应用提供新靶标。

## Abstract (en)

**Background and objective:**

Cyclic RNA (circRNA) is a new type of non-coding RNA (ncRNA) which is different from traditional linear RNA. More and more studies suggest that circRNA can be used as a biological marker of many malignant tumors and becomes a potential target for treatment. Therefore, searching for new molecular targets of lung adenocarcinoma from the circRNA will help to reveal the new mechanism of the occurrence and development of lung adenocarcinoma, and provide new ideas for clinical diagnosis and treatment. In this study, the biological function of circ_0007766, a highly expressed circRNA found in a screen of lung adenocarcinoma tissue, was verified and analyzed *in vitro*, so as to preliminarily explore the mechanism of circ_0007766 in promoting the proliferation of lung adenocarcinoma.

**Methods:**

The expression level of circ_0007766 in lung adenocarcinoma cells was detected by qPCR. Then siRNA was used to knock down the expression of circ_0007766. The effects of knockdown of circ_0007766 on proliferation, cell cycle and apoptosis of lung adenocarcinoma cells were detected by CCK8, scratch test, PI staining and Annexin V/PI double staining. In addition, the biological mechanism of circ_0007766 in lung adenocarcinoma was preliminarily studied by qPCR and Western blots.

**Results:**

The expression of circ_0007766 in lung adenocarcinoma cell lines was detected by qPCR. The expression of circ_0007766 was interfered in SPCA-1 cells. The proliferation and migration abilities of cells were inhibited. The cell cycle was arrested in G_0_/G_1_ phase, but the apoptosis was not affected. The deletion of circ_0007766 did not affect the expression of ERBB2, but influenced the mRNA and protein expression of Cyclin D1/Cyclin E1/CDK4.

**Conclusion:**

*In vitro* functional studies have shown that circ_0007766 may promote the proliferation and migration of lung adenocarcinoma cells. Further molecular mechanism studies have found that circ_0007766 can up-regulate the expression of Cyclin D1/Cyclin E1/CDK4, which are the key proteins of cell cycle, and thus promote the malignant proliferation of lung adenocarcinoma. From the perspective of circRNA, this study will provide new clues for the pathogenesis, development and prognosis of lung adenocarcinoma, and provide new target for clinical treatment.

近年来肺腺癌发病率逐步升高^[1]^，靶向药物成为肿瘤精准医学的先锋，诸如表皮生长因子受体（epidermal growth factor receptor, EGFR）、间变性淋巴瘤激酶（anaplastic lymphoma kinase, *ALK*）基因突变患者取得了较好的临床效果，但突变人群阳性率偏低，患者总体5年生存率仍徘徊在20%，表明肺腺癌发生发展的生物学行为及分子调控机制极其复杂。随着ENCODE计划的不断深入，极大地丰富了人们对于遗传信息网络的理解，让研究者们能更高效地了解细胞间相互调控的分子机制。circRNA作为非编码RNA家族中新兴成员之一，已经被证明在肿瘤的发生、发展中起着重要作用^[2, 3]^。

本研究前期通过芯片检测发现肺腺癌组织和癌旁组织中circ_0007766表达呈显著差异^[4]^，并在多个肺腺癌细胞株中进行体外验证，对circ_0007766在肺腺癌细胞的增殖、周期和凋亡等生物学功能进行体外水平研究和初步机制探索。

## 1 材料和方法

### 1.1 细胞和试剂

肺腺癌A549、SPCA-1、H1975及H1299细胞系均购于中国科学院上海生物化学以及细胞生物学研究所，人正常支气管上皮细胞BEAS-2B细胞购自于美国Sciencell公司。RPMI-1640和DMEM培养基购自美国Hyclone公司、胎牛血清购自美国Gibco公司；Trizol、siRNA和PARIS^TM^ Kit核浆分离试剂盒购自美国Invitrogen公司；CCK-8细胞增殖试剂盒和细胞周期试剂盒均购自南京凯基公司；Annexin V-FITC凋亡试剂盒购自上海贝博公司；SYBR Premix Ex Taq qPCR试剂盒购自中国大连Takara公司；CDK4、cyclin D1、cyclin E、p21和β-actin抗体均购自英国Abcam公司。

### 1.2 细胞培养

肺腺癌细胞均采用RPMI-1640培养基传代，具体配方为：RPMI-1640加入10%胎牛血清，100 U/mL青霉素以及100 mg/L链霉素。人正常支气管上皮细胞BEAS-2B细胞采用DMEM培养基传代，具体配方：DMEM培养液中加入10%胎牛血清，100 U/mL青霉素以及100 mg/L链霉素。培养箱条件设定为：温度37 ℃，5%CO_2_；每2天-3天更换新鲜培养基，细胞传代条件为汇合度大于3/4。

### 1.3 细胞增殖能力检测

细胞悬液接种于96孔板中，每组设5个复孔，约3, 000个细胞/孔/100 μL。将培养板放在培养箱中预培养（37 ℃，5%CO_2_）。每孔加入10 μL CCK8检测试剂，置于培养箱中孵育4 h。用酶标仪测定在450 nm处的吸光度，并测定各孔的OD值。

### 1.4 细胞周期检测

胰酶消化、离心后收集细胞，以预冷的无水乙醇（乙醇终浓度70%），吹打混匀，4 ℃固定过夜。离心并收集固定后的细胞，加入RNaseA于37 ℃水浴，最后加入PI常温下避光染色30 min，进行流式细胞仪上机检测，采用ModFit分析细胞周期分布。

### 1.5 细胞凋亡检测

胰酶消化、离心后收集细胞，在EP管中加入300 μL的1×Binding Buffer，制成单细胞悬液待标记检测，加入5 μL的Annexin V-FITC混匀后，置于避光环境下室温孵育15 min，再加入5 μL的PI染色标记悬浮细胞，流式细胞仪上机检测并分析细胞凋亡情况。

### 1.6 细胞划痕修复实验

用标记笔在6孔板背后用直尺均匀地划横线，大约每隔1 cm一道，横穿过孔，每孔至少穿过5条线。每孔中加入约5×10^5^个细胞，过夜后保证能铺满90%以上，第2天用移液枪头按照之前背面画的横线个尽量垂直在细胞培养板内划痕，用PBS漂洗细胞2次-3次，清除划掉的贴壁细胞，加入细胞培养液，放入37 ℃、5%CO_2_培养箱，培养，取样观察、拍照。

### 1.7 Real-time PCR检测

细胞总RNA采用Ttrzol试剂进行提取，分光分度仪测定总RNA浓度，经逆转录反应合成cDNA，以β-actin作为内参，ABI7500荧光定量PCR仪上SYBR Green Ⅰ染料检测各基因的表达情况。

### 1.8 RIP实验

收集细胞，加入细胞裂解液，每管加入磁珠悬液以及RIP冲洗液，vortex旋震荡。磁力分选后，弃去上清。RIP冲洗液重悬磁珠，加入配对抗体，室温静置30 min。磁力分选，弃上清。加入RIP缓冲液，离心免疫沉淀的小管，再次进行磁力分选，弃去上清。加入RIP冲洗液，vortex涡旋震荡后再次磁力分选，弃去上清。蛋白酶K缓冲液重悬上述磁珠-抗体复合物。孵育后将微管置于磁力架上，将上清液吸入一新的微管中，加入RIP缓冲液。每管加入苯酚、氯仿、异戊醇，vortex震荡后离心。吸取上层水相至新EP管中，加入氯仿，震荡后离心，吸取上层水相后移入新EP管中，加入盐溶液Ⅰ、盐溶液Ⅱ、沉淀增强剂、RNA-free无水乙醇后混合，-80 ℃过夜。离心后弃去上清，80%乙醇冲洗一次，再次离心后弃去上清，通风橱中晾干。RNA-free水重悬，用于逆转录以及qPCR分析。

### 1.9 Western blot蛋白印迹

胰酶消化、离心后收集细胞，加入细胞裂解液和蛋白膜抑制剂，提取细胞总蛋白，用BCA法定量，以10%SDS-聚丙烯酰胺凝胶电泳后转膜，用10%脱脂奶粉封闭，一次孵育一抗、二抗后，用Odyssey近红外荧光扫描成像系统扫膜分析。

### 1.10 统计分析方法

数据结果以均数±标准差（Mean±SD）计量，采用SPSS 17.0软件进行分析。多组间变量资料比较采用*ANOVA*进行检验，两组间变量资料比较采用*t*检验，以*P* < 0.05作为统计学显著性差异。

## 2 结果

### 2.1 常见肺腺癌细胞和临床样本中circ_0007766的表达情况

如图 1所示，通过qPCR检测，我们在肺腺癌常用4株细胞系中发现circ_0007766的表达，其表达水平均显著高于人支气管上皮样细胞BEAS-2B中circ_0007766的表达，提示circ_0007766在肺腺癌细胞中的表达具有普遍性。体外实验以circ_0007766表达水平最高的SPCA-1细胞（平均上调4.2倍，*P* < 0.01）和肺腺癌细胞A549为研究对象进行研究。此外，我们随机从江苏省肿瘤医院生物样本库中挑选了30例肺腺癌组织样本，进行qPCR的验证。结果发现，其中circ_0007766在肺腺癌组织中表达显著高于癌旁组织，平均上调2.76倍，差异表达水平显著（*P* < 0.05）。

**1:**
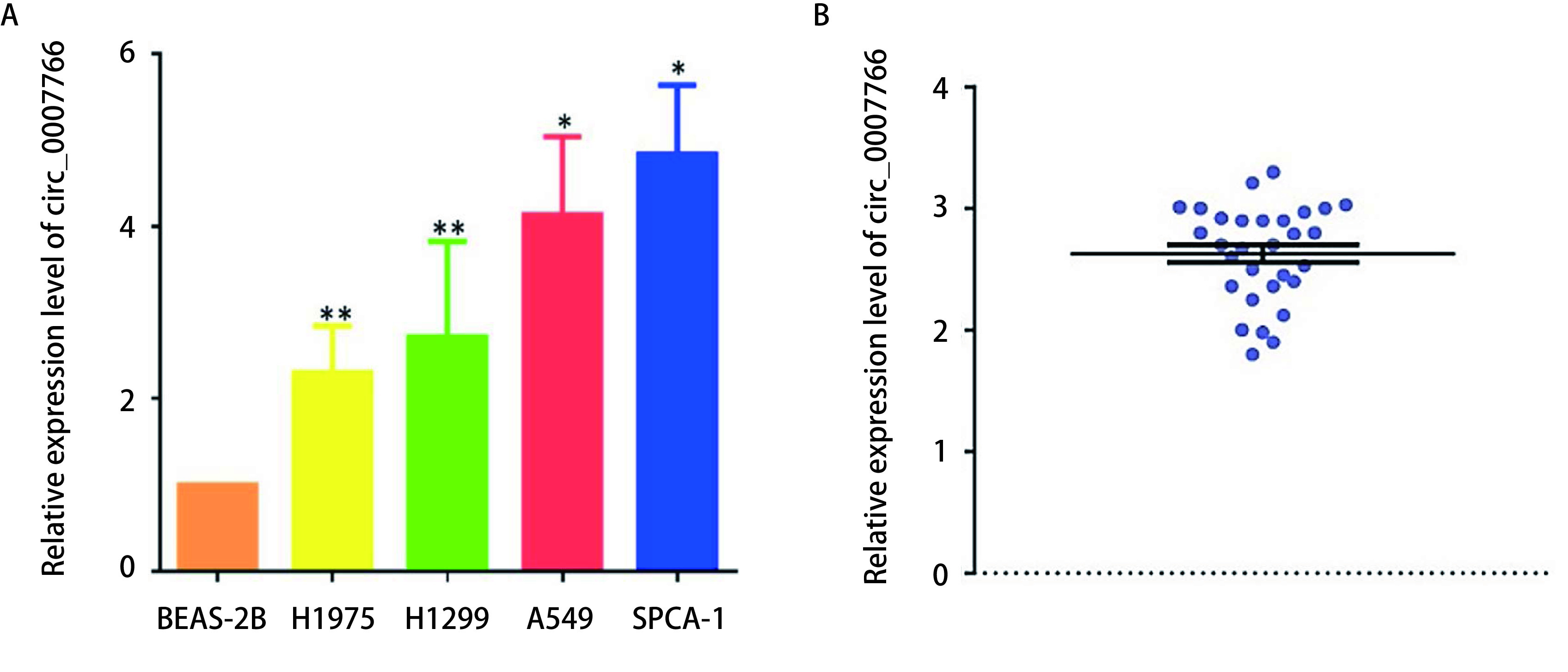
常见肺腺癌细胞系和临床样本中circ_0007766的表达水平。A：qPCR检测细胞系中内源性circ_0007766的表达情况，以正常支气管上皮细胞BEAS-2B为对照。（^*^P < 0.05，^**^P < 0.01）；B：在30例随机挑选的肺腺癌组织样本中进行qPCR的验证，结果发现circ_0007766差异表达显著，相较于癌旁组织平均上调2.76倍（*P* < 0.05）。 The expression level of circ_0007766 in common lung adenocarcinoma cell lines and clinical samples. A: The expression of endogenous circ_0007766 detected in normal bronchial epithelial cells BEAS-2B was detected as control (^*^P < 0.05, ^**^P < 0.01); B: The results of qPCR in 30 randomly selected lung adenocarcinoma tissues showed that circ_0007766 was significantly different in expression, which was 2.76 times higher than that in adjacent tissues (*P* < 0.05).

### 2.2 circ_0007766可以促进SPCA-1和A549细胞的增殖能力

采用siRNA干扰SPCA-1和A549细胞中circ_0007766的表达，通过CCK-8实验研究其增殖能力的变化。分别检测0 h，24 h，48 h，72 h和96 h的OD值，如图 2所示。相比较于NC组，转染si_circ_0007766后SPCA-1和A549细胞中增殖速率均显著降低。上述结果表明降低circ_0007766的表达对SPCA-1和A549细胞的增殖具有抑制的作用。

**2:**
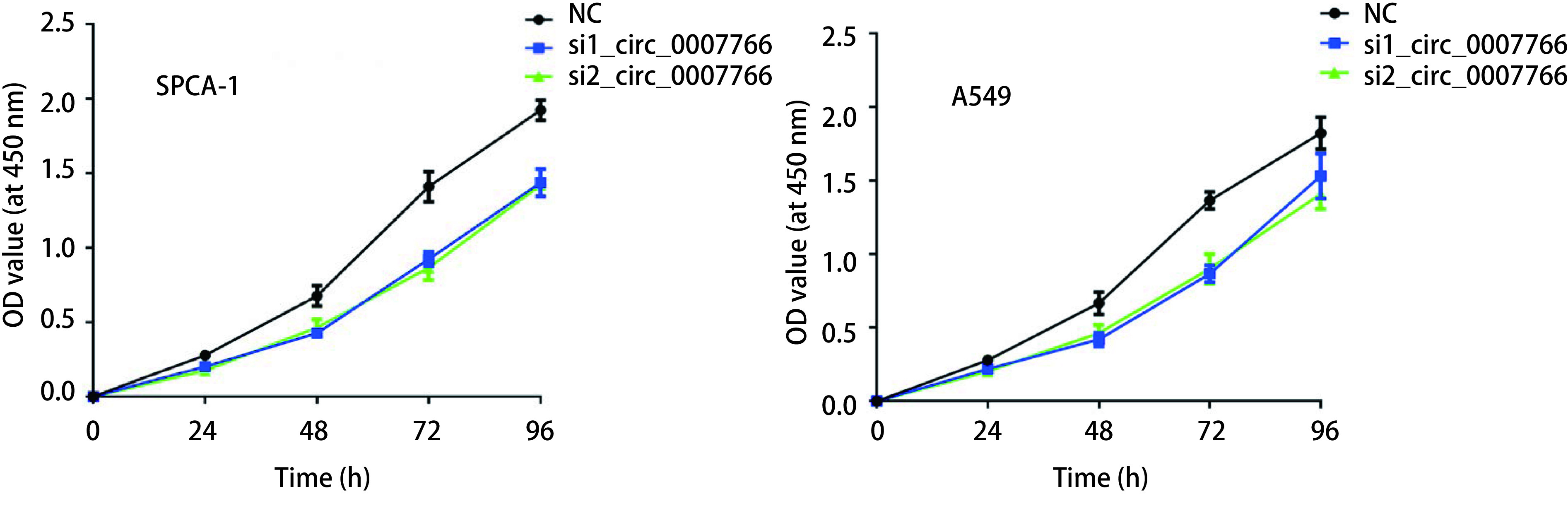
circ_0007766对肺腺癌细胞增殖能力的影响。CCK8实验检测细胞转染后0 h、24 h、48 h、72 h及96 h时的细胞活力。每组设置5个复孔，用酶标仪测定在450 nm处的吸光度，并测定各孔的OD值。 Effect of circ_0007766 on cell proliferation of lung adenocarcinoma cell lines. CCK8 assay was carried out to detect the cell viability of cells transfected by siRNA for 0 h, 24 h, 48 h, 72 h and 96 h. Five compound pores were set in each group. The absorbance at 450 nm was measured by enzyme labeling instrument, and the OD value of each pore was determined.

### 2.3 circ_0007766可以促进SPCA-1和A549细胞的划痕修复能力

SPCA-1和A549细胞经转染siRNA沉默circ_0007766后，采用划痕修复实验研究其迁移运动能力是否受到影响。分别观察0 h、12 h和24 h的细胞划痕修复情况（图 3），结果按照划痕两侧细胞结合度百分比计算，相比较于对照组，转染si_circ_0007766约24 h后SPCA-1和A549细胞划痕修复能力显著降低。上述结果表明降低circ_0007766的表达可以抑制SPCA-1和A549细胞的划痕修复能力。

**3:**
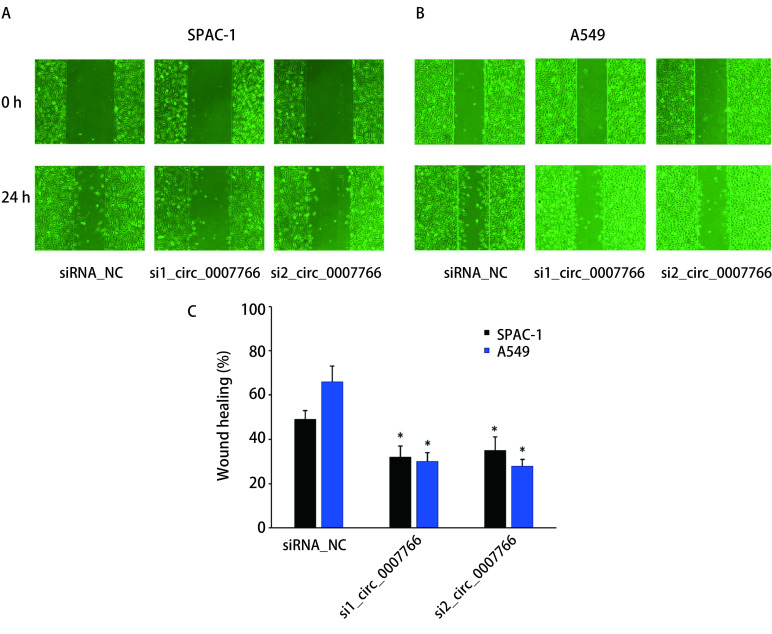
细胞划痕修复实验及统计分析。A：细胞划痕修复实验检测SPAC-1和A549细胞迁移的能力；B：相较于siRNA对照组，转染circ_0007766的siRNA后细胞划痕修复能力下降（^*^P < 0.05）。 The cell scratch repair experiment and statistical analysis. A: The cell scratch repair experiment were used to measure cell migration capacity of SPAC-1 and A549 cells; B: The ability of cell scratch repair after transfection of siRNA of circ_0007766 decreases compared with siRNA control group (^*^P < 0.05).

### 2.4 circ_0007766对SPCA-1和A549细胞的细胞周期的影响

SPCA-1和A549细胞经转染siRNA沉默circ_0007766后，采用PI单染方法检测细胞周期分布的影响（图 4）。相比较于对照组，转染si_circ_0007766约48 h后SPCA-1和A549细胞G_0_期/G_1_期周期比例明显增多。上述结果表明沉默circ_0007766后的SPCA-1和A549细胞受阻于G_0_期/G_1_期。

**4:**
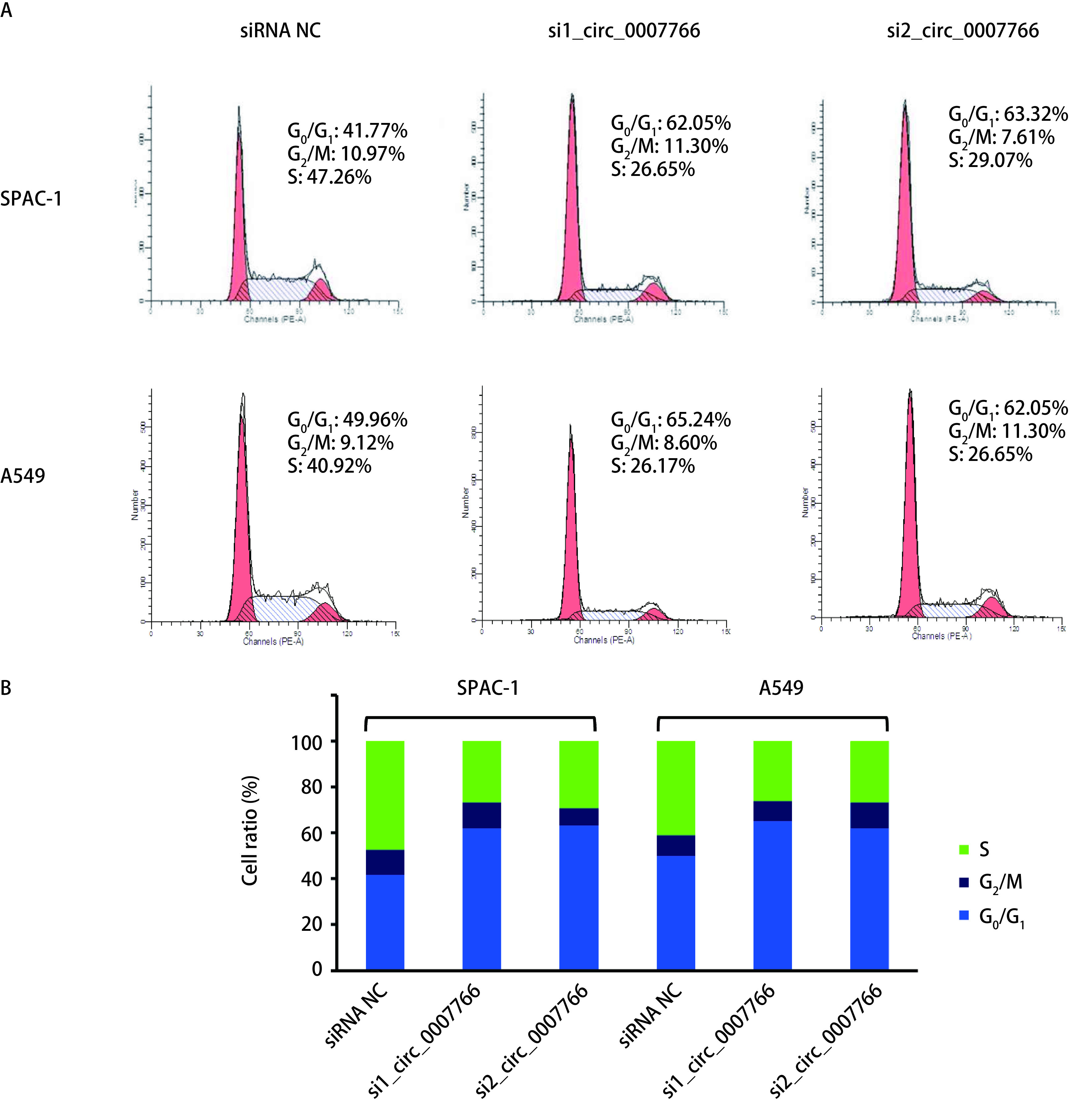
circ_0007766对SPCA-1和A549细胞周期的影响及统计分析。A：各组细胞固定后加入PI染色，进行流式细胞仪上机检测，用细胞周期拟和软件ModFit分析。分析时使用FL2-w和FL2-A模式；B：实验组细胞受阻于G_0_/G_1_期，该期细胞比例显著升高（^*^P < 0.05, ^**^P < 0.01）。 Effect of circ_0007766 on cell cycle of SPCA-1 and A549 cells and statistical analysis. A: Cells in each group were fixed and stained with PI. Flow cytometry was used to detect the cell cycle; B: The results were analyzed by cell cycle simulation software ModFit. FL2-w and FL2-A patterns were used. Cells in the experimental group were arrested in G_0_/G_1_ phase, and the proportion of cells in this phase was significantly increased (^*^P < 0.05, ^**^P < 0.01).

### 2.5 circ_0007766对SPCA-1和A549细胞凋亡的影响

采用Annexin V-FITC双染标记进行细胞凋亡实验（图 5），相比较于NC组，转染si_circ_0007766后SPCA-1和A549细胞的凋亡比例并无显著差异。

**5:**
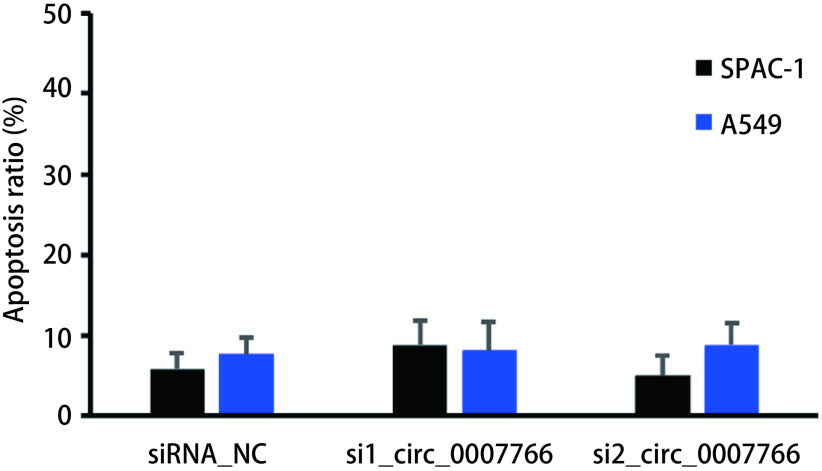
circ_0007766对SPCA-1和A549细胞凋亡的影响及统计分析。A：各组细胞接受AnnexinV和PI染色后采用流式细胞术检测凋亡细胞比例；B：相较于对照组，转染si_circ_0007766后SPCA-1和A549细胞的凋亡比例并无显著差异。 Effect of circ_0007766 on apoptosis of SPCA-1 and A549 cells and statistical analysis. A: The percentage of apoptotic cells was detected by flow cytometry after AnnexinV and PI staining; B: Compared with the control group, there was no significant difference in the percentage of apoptotic cells of SPCA-1 and A549 after transfection of si_circ_0007766.

### 2.6 circ_0007766并非通过调控亲本基因*ERBB2*的表达发挥作用

通过circBase网站（http://www.circbase.org/）、UCSC（http://genome.ucsc.edu/）等数据库检索表明circ_0007766由*ERBB2*基因的6号-10号外显子环化形成，剪切长度为676 bp，属于外显子来源circRNA。ERBB2又名Her2，是表皮生长因子受体家族成员，作为一种原癌基因其在细胞表面高表达代表肿瘤恶性程度更高，细胞增殖能力强。我们推测circ_0007766可能通过顺式作用调控其亲本基因*ERBB2*发挥作用。体外实验发现相较于对照组，沉默circ_0007766后SPCA-1细胞中ERBB2的mRNA及蛋白表达均无显著变化（图 6）。

**6:**
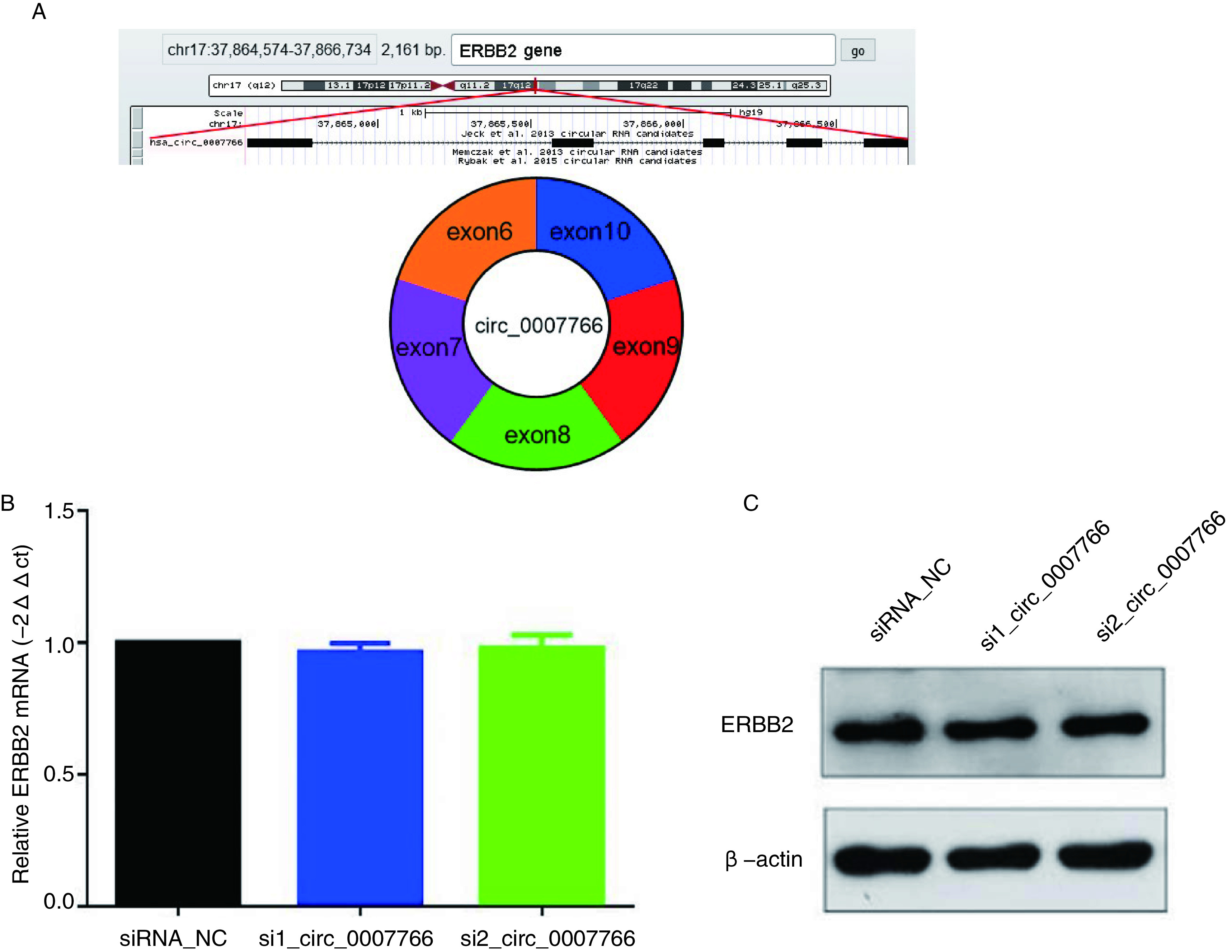
circ_0007766沉默后肺腺癌细胞中亲本基因ERBB2的表达。A：circ_0007766由母基因ERBB2的6至10号外显子环化形成，剪切长度为676 bp，属于外显子来源circRNA；B：qPCR检测siRNA转染后细胞中亲本基因*ERBB2*的表达水平；C：Western blot检测siRNA转染后细胞中ERBB2蛋白的表达水平。 Expression of ERBB2 in lung adenocarcinoma cells after circ_0007766 silencing. A: Circ_0007766 is formed by cyclization of exons 6 to 10 of the parent gene *ERBB2*, with a shear length of 676 bp. It belongs to exon-derived circRNA; B: The expression level of ERBB2 was detected by qPCR after siRNA transfection; C: Western blot were used to detect the expression of ERBB2 protein in siRNA transfected cells.

### 2.7 circ_0007766显著下调Cyclin D1/Cyclin E1/CDK4的mRNA表达

由于沉默circ_0007766表达后肺腺癌细胞增殖受阻，细胞周期受阻于G_0_期/G_1_期，为了验证这一推测，我们以体外circ_0007766表达丰度最高的SPCA-1细胞为模板，沉默circ_0007766表达后SPCA-1细胞中Cyclin D1、Cyclin E1及CDK4 mRNA表达显著下调，而P21基因mRNA表达无显著变化（图 7）。

**7:**
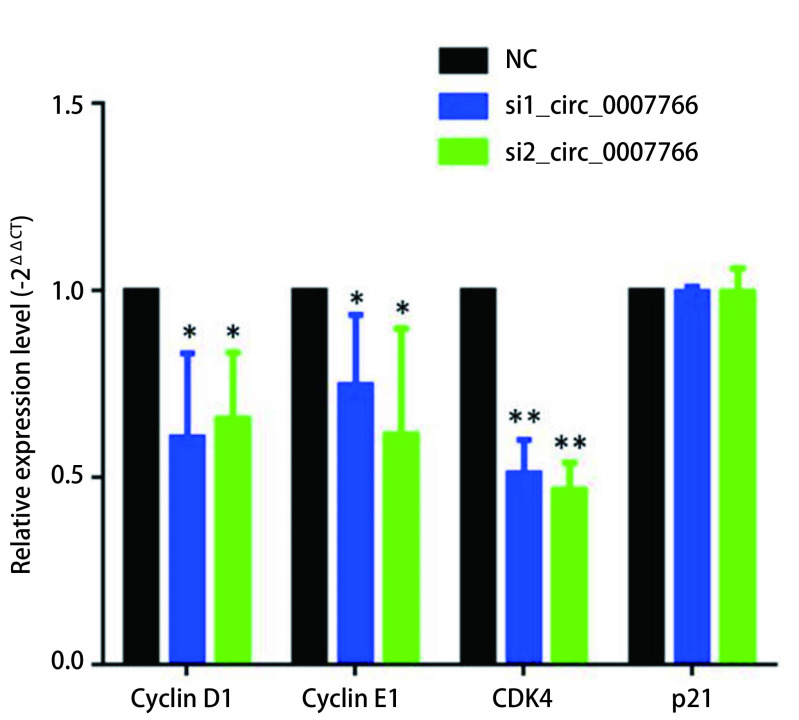
细胞周期关键基因*Cyclin D1*/*Cyclin E1*/*CDK4* mRNA表达（^*^P < 0.05，^**^P < 0.01）。 mRNA expression of key genes of cell cycle including *Cyclin D1*, *Cyclin E1*, and *CDK4* (^*^P < 0.05, ^**^P < 0.*P* < 0.01).

### 2.8 circ_0007766与eIF4A3蛋白具有结合能力

eIF4A3蛋白是真核生物延长翻译因子家族成员之一，作为一种RNA结合蛋白可与靶mRNA结合，并通过无义介导的mRNA降解途径（nonsense-mediated mRNA decay, NMD）在转录后水平抑制基因表达^[5]^。我们进一步运用生物信息学方法预测（https://circinteractome.nia.nih.gov），发现circ_0007766与eIF4A3蛋白有多达8个结合位点。于是我们进一步进行RIP实验，验证circ_0007766与eIF4A3蛋白具有结合能力（图 8）。

**8:**
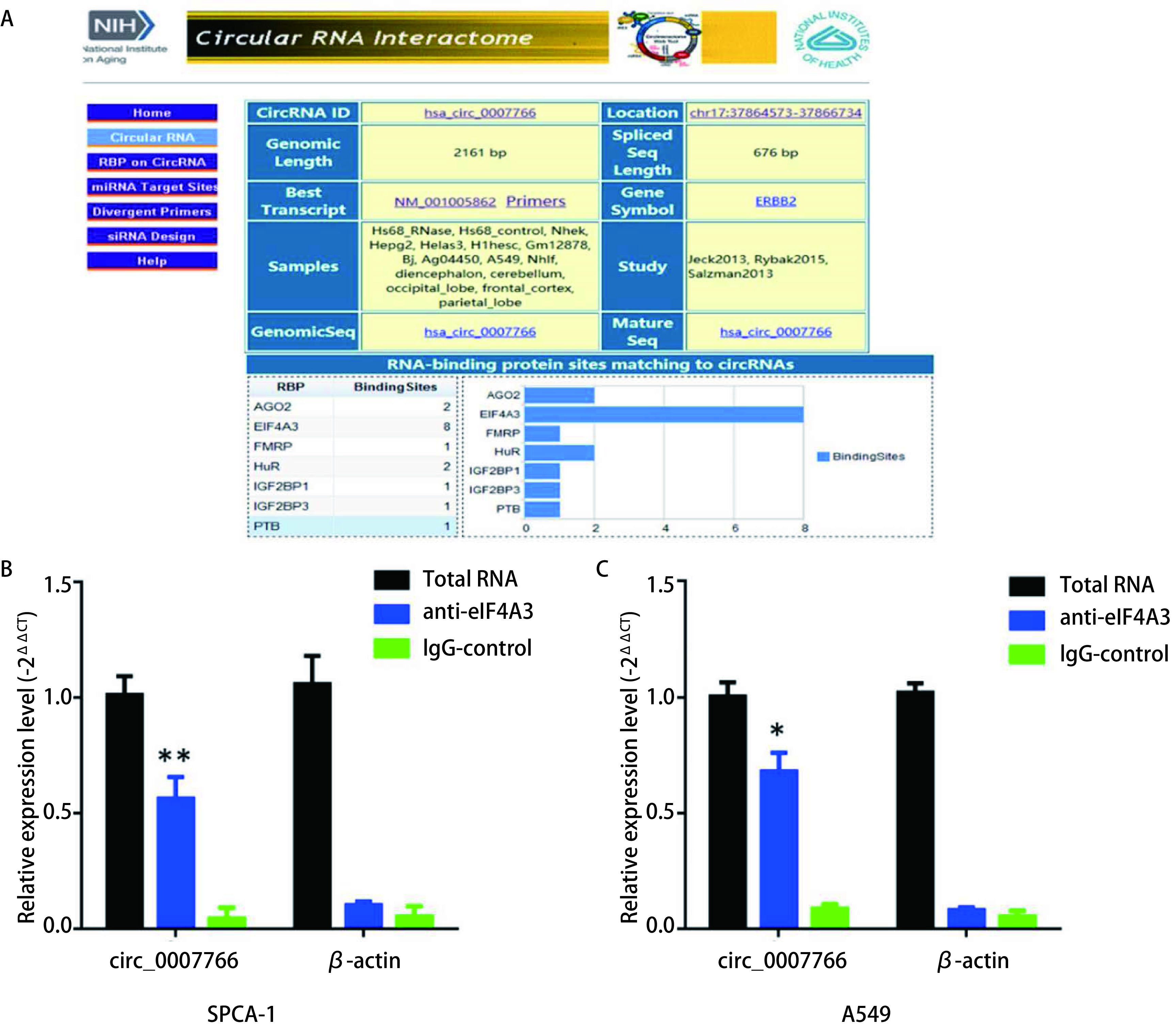
circ_0007766与eIF4A3蛋白结合能力生物信息学预测及RIP实验。A：生物信息学预测发现circ_0007766与eIF4A3蛋白有多达8个结合位点，在所有结合蛋白中结合位点数最多；B：以IgG为对照，qPCR检测eIF4A3抗体所结合的RNA及circ_0007766表达水平。 Bioinformatics Prediction and RIP assay for the binding ability of circ_0007766 to eIF4A3. A: Bioinformatics predictions revealed that circ_0007766 and eIF4A3 had eight binding sites, with the largest number of binding sites among all binding proteins; B: Using IgG as control, qPCR was used to detect the expression level of RNA and circ_0007766 bound by eIF4A3 antibody.

### 2.9 circ_0007766可能通过影响eIF4A3下调细胞周期蛋白Cyclin D1，Cyclin E1及CDK4的表达

文献报道，结肠癌中细胞周期关键蛋白Cyclin D1、Cyclin E1及CDK4是eIF4A3的下游靶标^[6]^，在肺腺癌中是否具有类似机制尚不得而知。在沉默circ_0007766表达后检测发现，细胞周期关键蛋白Cyclin D1/Cyclin E1/CDK4的表达明显下调，而对P21蛋白的表达无显著影响。我们结合生物信息学分析并推测，circ_0007766可能通过结合eIF4A3蛋白在转录后水平调控细胞周期相关基因表达，进而调控肺腺癌增殖。在沉默eIF4A3蛋白表达后下游靶基因Cyclin D1/Cyclin E1/CDK4的表达效应可以通过沉默circ_0007766得到部分逆转，而P21蛋白无显著变化（图 9）。以上结果表明，circ_0007766可能是通过招募eIF4A3蛋白调控细胞周期关键蛋白Cyclin D1/Cyclin E1/CDK4的表达。

**9:**
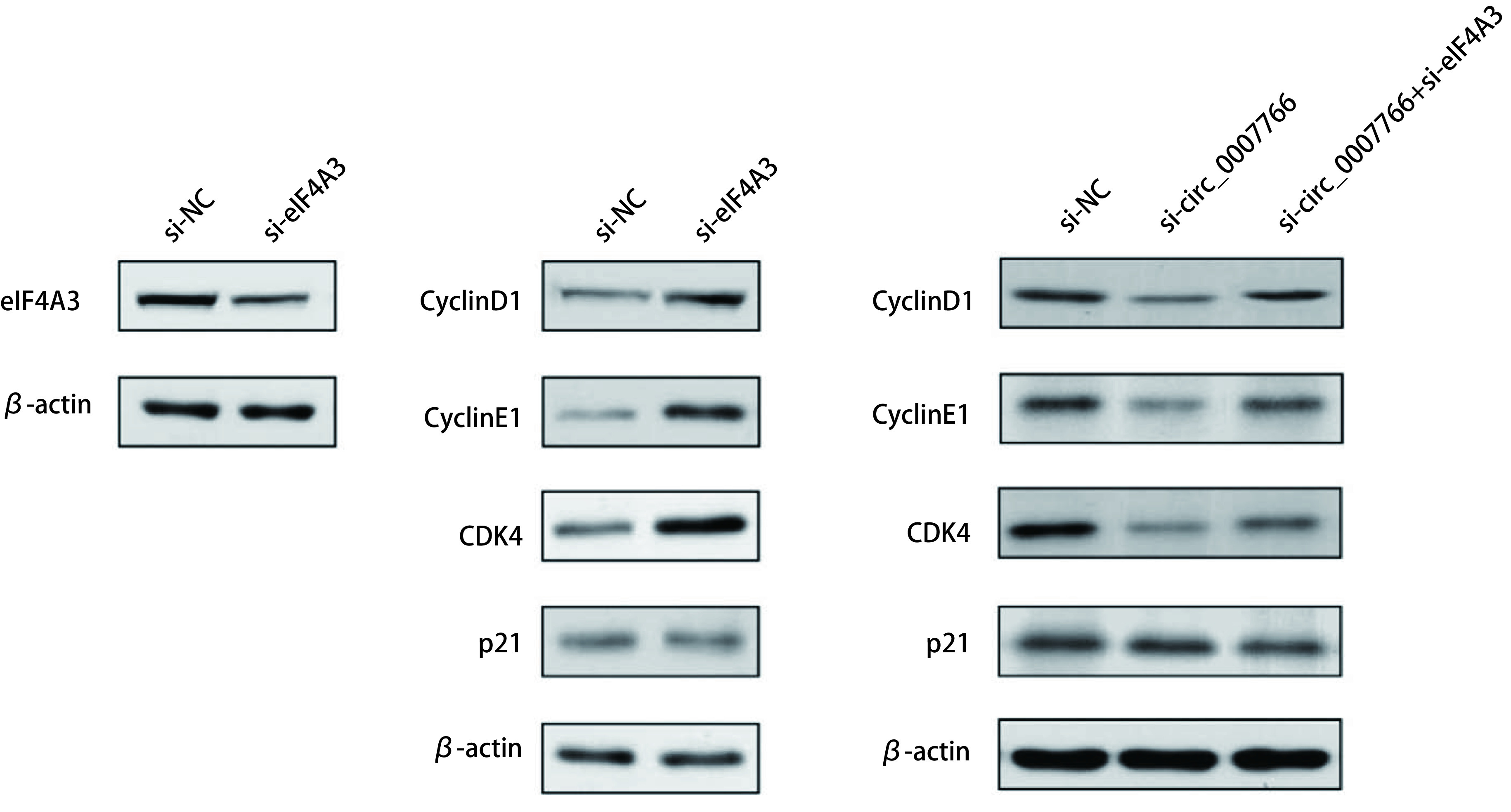
沉默circ_0007766和/或eIF4A3后Cyclin D1/Cyclin E1/CDK4的蛋白表达 Protein expression of Cyclin D1/Cyclin E1/CDK4 in cells silencing circ_0007766 or eIF4A3

## 3 讨论

circRNA是区别于传统线性RNA的一类新型RNA，具有闭合环状结构，大量存在于真核转录组中。circRNA通常由宿主基因外显子或内含子剪切环化而成；由于其在结构上封闭成环，对RNA酶不敏感，因而较线性RNA更稳定，具备理想肿瘤标志物的天然优势^[7]^。越来越多的证据表明circRNA与肿瘤的发生、发展密切相关，为我们提供了理解肿瘤生物学特征的新视角。目前肺腺癌相关circRNA的研究处于起步阶段，我们通过体外沉默circ_0007766表达的技术，针对SPCA-1和A549细胞设计特异性的siRNA，观察并验证si_circ_0007766的体外效应。另外，为观察circ_0007766对肺腺癌细胞增殖能力的影响，我们通过CCK8和PI单染、实验来进行检测。实验结果提示沉默circ_0007766表达后肺腺癌细胞增殖能力显著下降，细胞周期受阻于G_0_/G_1_期。

细胞凋亡与坏死不同，细胞凋亡是一个主动过程，涉及一系列基因的激活、表达及调控等过程；是肿瘤细胞为更好地适应生存环境而主动争取的一种死亡过程，在肿瘤的发生发展过程中具有重要的作用^[8]^。为研究circ_0007766对肺腺癌细胞凋亡功能的影响，我们利用Annexin V-FITC/PI双染流式细胞检测，结果表明沉默circ_0007766表达后的肺腺癌细胞凋亡并未有显著变化。综上所述，我们研究认为circ_0007766主要通过促进细胞的增殖参与肺腺癌的发生、发展，是一种潜在的“促癌因子”。

在探索circ_0007766的机制研究中，我们通过查阅文献，发现目前外显子circRNA主要通过三种机制来调控基因表达^[9, 10]^：①调控亲本基因；②ceRNA机制；③竞争性结合蛋白调控靶基因。通过进一步的研究，我们发现circ_0007766可能是通过结合蛋白调控靶基因的方式发挥生物学作用。

最近有报道^[11-13]^显示，circRNA可以通过结合PES1蛋白调控核糖体成熟和转录因子FOXO3诱导细胞凋亡和周期阻滞。我们推测circ_0007766是否具有类似的调控机制，遂将研究重点转到circRNA-蛋白结合调控基因表达这一方向。首先通过生物信息学分析，我们发现eIF4A3蛋白在circ_0007766能够结合的蛋白质中结合位点最多，我们将其作为重点研究对象。eIF4A3蛋白作为eIF4A真核翻译起始因子家族成员之一，可与靶mRNA结合，并通过无义介导的mRNA降解途径在转录后水平抑制基因表达^[14, 15]^。例如eIF4A3可与细胞周期调控关键基因*CDK4*和凋亡相关基因*Bcl-x*等癌基因mRNA结合在转录后水平下调其表达从而抑制肿瘤恶性进展^[6, 16]^。我们针对circ_0007766和eIF4A3蛋白进行RIP实验验证，结果表明circ_0007766可与eIF4A3蛋白结合。

在功能学研究中，我们发现circ_0007766主要影响肺腺癌细胞的增殖功能，沉默其表达后细胞周期明显受阻于G_0_期/G_1_期，引起该周期相关蛋白Cyclin D1，Cyclin E1、CDK4及P21表达显著下降。基于以上结果，我们推测这种效应的结果是circ_0007766与eIF4A3蛋白介导NMD途径实现的。随后我们进行实验验证了这一推测，设计circ_0007766与eIF4A3蛋白交叉沉默分组实验，结果发现沉默circ_0007766表达引起的周期蛋白Cyclin D1/Cyclin E1/CDK4表达下降的效应可被沉默eIF4A3蛋白所拯救，而对P21蛋白的表达影响不大。

综上所述，本研究从circRNA这一新视角出发，通过对肺腺癌与癌旁组织样本的高通量芯片筛查获取差异表达circRNA，结合临床病理及随访资料发现circ_0007766与肺腺癌患者TNM分期及预后呈显著正相关。体外功能实验发现沉默circ_0007766可抑制肺腺癌细胞的增殖，细胞周期受阻于G_0_期/G_1_期。分子机制研究表明circ_0007766主要通过影响eIF4A3蛋白功能，从而上调周期蛋白Cyclin D1/Cyclin E1/CDK4的表达，进而促进肺腺癌细胞的增殖。本研究可为肺腺癌发生发展机理及预后判断提供新线索，为临床治疗应用提供新靶标。

## References

[1] Zhang RF, Zhang Y, Wen FB (2016). Analysis of pathological types and clinical epidemiology of 6, 058 patients with lung cancer. Zhongguo Fei Ai Za Zhi.

[2] Meng S, Zhou H, Feng Z (2017). CircRNA: functions and properties of a novel potential biomarker for cancer. Mol Cancer.

[3] Kristensen LS, Hansen TB, Venø MT (2018). Circular RNAs in cancer: opportunities and challenges in the field. Oncogene.

[4] Qiu M, Xia W, Chen R (2018). The circular RNA circPRKCI promotes tumor growth in lung adenocarcinoma. Cancer Res.

[5] Corinna G, Yeo GW, Stone ME (2007). The EJC factor eIF4AIII modulates synaptic strength and neuronal protein expression. Cell.

[6] Dong H, Xu G, Meng W (2016). Long noncoding RNA H19 indicates a poor prognosis of colorectal cancer and promotes tumor growth by recruiting and binding to eIF4A3. Oncotarget.

[7] Vicens Q, Westhof E (2014). Biogenesis of Circular RNAs. Cell.

[8] Wong RS (2011). Apoptosis in cancer: from pathogenesis to treatment. J Exp Clin Cancer Res.

[9] Ling-Ling C, Li Y (2015). Regulation of circRNA biogenesis. Rna Biol.

[10] Huang S, Yang B, Chen BJ (2017). The emerging role of circular RNAs in transcriptome regulation. Genomics.

[11] Holdt LM, Stahringer A, Sass K (2016). Circular non-coding RNA ANRIL modulates ribosomal RNA maturation and atherosclerosis in humans. Nat Commun.

[12] Du WW, Fang L, Yang W (2016). Induction of tumor apoptosis through a circular RNA enhancing Foxo3 activity. Cell Death Differ.

[13] Du WW, Yang W, Liu E (2016). Foxo3 circular RNA retards cell cycle progression via forming ternary complexes with p21 and CDK2. Nucleic Acids Res.

[14] Giorgi C, Yeo GW, Stone ME (2007). The EJC factor eIF4AIII modulates synaptic strength and neuronal protein expression. Cell.

[15] Conti E, Izaurralde E (2005). Nonsense-mediated mRNA decay: molecular insights and mechanistic variations across species. Curr Opin Cell Biol.

[16] Michelle L, Cloutier A, Toutant J (2012). Proteins associated with the exon junction complex also control the alternative splicing of apoptotic regulators. Mol Cell Biol.

